# The Probable Usefulness of the Silicate Cements

**Published:** 1909-06-15

**Authors:** W. B. Dunning

**Affiliations:** New York, N. Y.


### THE PROBABLE USEFULNESS OF THE SILICATE CEMENTS.

BY W. B. DUNNING, D.D.S., NEW YORK, N. Y.

Read before the New York Odontological Society, at its regular monthly meeting, November 17, 1908.

It is the purpose of this paper to develop a discussion on the merits and the shortcomings of the new silicate cements. The writer can speak only from experience of two or three years in the use of this new filling material, which experience unfortunately nearly covers the length of time that this material has been available in dental practice. No one is yet prepared to speak in an authoritive way of its practical value as a tooth-saver, since it has not yet stood the test of time; but we all, perhaps, have been so impressed with its probable good qualities as to feel that a comparison of notes should be made, as a point of departure for further observations.

It is to be regretted that the manufacturers of the principal silicate cements have adhered to the ancient trade custom of keeping secret the exact formulae of their preparations, regardless of the fact that the patent records are open to the public and that their preparations may be analyzed. No purpose of protection is in this way served against individuals who may be bent upon infringement, while the conscientious operator, from lack of time or knowledge of facilities, fails to satisfy his very proper desire to know the nature of what he is using. In consequence of this short-sighted policy, many-would be consumers have hesitated to freely use these preparations.

In reply to a letter to the L. D. Caulk Company requesting information concerning their preparation Translux, I have received the following, which without referring specifically to their own product, is interesting and valuable as dealing with the subject at large:

Your letter of inquiry in regard to silicate cements was received several days ago, but owing to a rush of business and the technical nature of the answer required, it has been unavoidably delayed. We trust the following will cover the points that you have in mind.

The powder as applied in dentistry consists of compound silicates of lime and alumina, with aluminates, modified by addition of other basic silicates which the manufacturers may determine advantageous in production of desired modifying effects.

The liquid is a solution of orthophosphoric acid, containing aluminum phosphate and ingredients used to control rapidity of action upon the powder.

Mixing the powder with the acid solution a chemical reaction is produced, during which any particles of oxid present are seized upon by the acid and converted into insoluble phosphates, leaving the silicates in a hydrated condition, and binding the mass into a dense body, or artificial stone, like in its properties.

After the time allowed for complete chemical combination—or "setting"—this material will indefinitely remain hard in a dry state, and in a wet state resists action of most solvents, excepting *strong* acids or alkalies.

Possessing these properties, and showing but slight expansion or contraction under normal conditions, viz, in moist state, it naturally follows that in the mouth it possesses lasting qualities, particularly as it is capable of high polish, thus further enhancing its resistance to the action of solvents.

All silicate cements have virtually been based upon similar materials, and reasons for failure are so numerous and varied as to defy classification.

A few principal causes are given:

Lack of accuracy on the part of the manufacturer in calculating percentages of ingredients to form a product in which the chemical bonds were properly satisfied. Result: Excess of one or more materials, which entering uncombined into the plastic mass necessarily introduced an elementof weakness, interfered with the correct action of acid solution of a given strength, and resulted in the presence of free soluble matter or material in such shape that, lacking affinity or combination with other bases, it could be washed out or would break away.

The use of too much flux in the effort to reduce raw materials at low temperatures, resulting in the introduction of free soluble matter; the lack of knowledge regarding degrees of heat and length of time required to obtain desired results, and failure to recognize the absolute necessity for careful tests to demonstrate absence of error during every step in the process of manufacture.

On the part of the profession, lack of proper manipulation, not recognizing the fact that the silicate cements cannot be handled successfully without special preparation of the cavity as for gold or amalgam; or, in other words, that these cements cannot be used with the same indifference to detail as are the oxyphosphate cements, as none of the silicates after having become hard retain the adhesiveness of an oxyphosphate, but rather depend upon the shape of the cavity to hold them in position.

The powder of an oxyphosphate, if properly prepared, is composed essentially of true oxids, which upon combining with phosphoric acid are converted into oxyphosphates.

The powder of a silicate cement is composed—(theoretically)— entirely of silicates or their combinations, though there may be *traces* of oxids, or, upon exposure, hydrates or carbonates, but only in the most *minute* quantities.

Silicates, as the name indicates, are compounds of silica with various elements or bases. Under favorable conditions, silica acts as an acid, and at high temperature effects many combinations. While there are exceptions in which it enters into volatile conditions, as a rule we obtain vitreous masses —such natural products as rock crystal, various spars, and many of the semi-precious stones, and artificially, glass, porcelain, etc.

These combinations, as a rule, are practically insoluble substances, which yield only under favorable conditions to the action of strong acids or alkalies, and then only through special treatment.

The chemical action of silica is so complex, it being analogous to carbon in this respect, that while a general knowledge of its actions is held, there are yet many fine points for chemical research. Artificial silicates, after grinding and exposure, might, as stated, contain trace of oxids with which the phosphoric acid would combine, but not sufficient to do more than aid ever so slightly in the binding, and it could not by any strength of the imagination be considered as an "oxyphosphate cement."

It is more than likely that the gelatinizing of the finely divided silicates through action of the acid is responsible for the direct cementation, or, in other words, the freeing and re-arrangement of silicic acid; they may therefore be called true silicate cements.

In silicates, if properly prepared, even after the powder has been converted into a cement through action of the liquid, any trace of phosphates present would be of an insoluble character.

Tests on slabs of silicate cements in four per cent. solutions of hydrogen sulfid or ammonium sulfid, and allowed to remain two weeks, or until the solution has been decomposed and the free sulfur deposited, have failed to show any marked discoloration, and such tests are surely more severe in this regard than could possibly occur in the oral cavity.

Sulfuretted conditions exist in secretions of all mouths to a greater or less degree, and it is logical to conclude that if this were the cause for discoloration such fault would be general, and not confined to isolated cases, as is the fact. In light of the knowledge presented it would seem we must look farther for the *one* fault—discoloration.

Granting the good points which silicates *do possess*, it is surely to the advantage of the profession to give them an unbiased trial and not jump to unfavorable conclusions without direct and convincing evidence.

I am indebted to Mr. Pinches of this city, representing Ascher's Artificial Enamel, for much information, short of the precise formula, concerning the nature of his preparation. As this compound was the first practicable silicate cement to be put into our hands, and as my brief experience has been derived almost entirely from its use, I shall speak of it as a type, though in no way discriminating against the dozen or more other preparations now on the market, nor inferring in any way that Ascher's is, or is not, the best material of this kind for our purpose.

The inventor, the German chemist Steenbock, in his "Specification forming part of letters patent No. 771,184, dated September 27, 1904," speaks as follows regarding the nature of this material:

My invention relates to a process of manufacturing vitreous cement which substantially consists in mixing phosphoric acids or their acid salts in solution with beryllium compounds decomposable by or reacting with the same. The product thus obtained, which possesses excellent cementing qualities and is particularly suitable for plugging teeth, exhibits an extremely high transparency, this being quite a new feature with cements produced by the cold method. Transluscent cements have always been obtained by a melting process, but this process is too inconvenient and difficult to be employed, for instance, in the case of pluggings for teeth besides being very expensive and frequently attended by failure.

The material produced by my aforesaid process in a convenient manner is quite vitreous, slightly opalescent, and has an exceedingly delicate bluish-white tint, by reason of which it is especially adapted to be used for plugging delicately transluscent teeth, as a cement for pearls, fine porcelain, and the like. In addition to the aforesaid high transparency, which is a new property in cements of the kind, and the deep luster due to such property, the cement manufactured by my process constitutes a new chemical compound differing materially from all cements of a cognate character. In contradistinction to the other cements, which are formed chiefly of zinc, calcium, and magnesium phosphates, this cement consists almost exclusively of pure beryllium compounds.

I am aware that cements are used to the acids of which beryllium oxid has been added, but this addition is only designed for delaying the hardening, and may in this function be replaced by magnesium oxid. It has no determining influence upon the composition of the hardened cement, which does not differ from that of other cements. The quantity added is so slight that the cement produced does not by any means consist of beryllium compounds, such as is the case with the cement made by my process.

A convenient mode of carrying this process into effect is as follows: A deposit formed by precipitating a solution of basic beryllium nitrate. $Be(NO_3)_2 2BeO$, with sodium silicate, $Na_2SiO_3$, after it had remained for some length of time under water, is filtered, carefully washed, dried, and slightly calcined. The preparation obtained—the emperic formula of which is $3BeOSiO_2$—is finely ground and used by itself, or, if greater consistence be required, is intimately mixed with glass or pure clay. The powder is carefully triturated with an approximately 52 per cent. orthophosphoric acid in which aluminum phosphate containing a little zinc phosphate or strontium phosphate is dissolved almost to saturation. In this manner a plastic material is obtained which in a short time sets to form a cement exhibiting the properties hereinbefore set forth. It is supposed that in the hardening the acid liquid withdraws beryllium oxide from the basic beryllium silicate, leaving a hydrated silicate behind and forming phosphate.

From this technical description it is to be gathered that the liquid consists of a nearly saturated solution of aluminum, zinc, and strontium phosphates in 52 per cent. orthophosphoric acid, and that the powder consists of the combination of beryllium nitrate with sodium silicate. These two elements are supplied for our use, and their combination through proper mixing, produces the elaborate compound to be discussed. Ascher's enamel bases its claim to permanence on the insolubility of beryllium in the fluids of the mouth, this mineral comprising 24 per cent. of the powder.

It will be seen that the name "chemical" or "plastic" porcelain has very fitly been used to describe the nature o the silicate cement. A filling of the same contains many of the constituents of fused porcelains, but the compound has been made at ordinary temperature by the union of a powder and a liquid, instead of, as in the case of true porcelain, the union of base and flux at a high temperature. We are cautioned by the manufacturers that this "chemical porcelain" can never equal fused porcelain in strength and durability.

The physical characteristics of silicate cement, when set resemble those of porcelain. The same degree of translus-cency is not obtained and the cement does not retain the glaze beyond a certain length of time, My observations are based principally upon my own fillings, inserted during the past three years, and some allowance upon these points should probably be made for error in manipulation. Further than the loss of the glaze, I have not seen evidences of wasting by solution.

### FIELD OF USEFULNESS.

The silicate cement seems especially adapted to the many small cavities in the incisor and bicuspid regions where metal would be unsightly, and where the retention of minute porcelain inlays would be uncertain. These labial, buccal, or approximal cavities are not exposed to stress of wear, but subject to the chemical influence of mucous secretions and other fluids of the mouth. An insoluble plastic porcelain in the region named, even though lacking somewhat in edge strength, but possessing the other qualities of porcelain, seems almost ideally suited for our purpose. Many of the minute approximal cavities, where much cutting would be necessary for inlays, may be perfectly filled with this substance at a minimum loss of sound tooth-structure, and aside from this consideration, the patient will be saved much of the fatigue and pain of the larger operation. It is also a great advantage not to be hampered with the usual film of opaque cement, which with the inlay interferes with the transmission of light, and by gradual solution, leaves the porcelain edges more or less unsupported and liable to chip.

It has been my practice heretofore to confine the use of this material to the small cavities mentioned, partly because I doubted its ability to withstand a crushing stress, and partly because in a large cavity in which a well-constructed porcelain inlay may be used, it is possible with the inlay to secure the nice blending of shades necessary at different parts of the tooth. In talking recently with many excellent operators,

I find that they have not hesitated to fill large compound cavities and restore contours which have successfully withstood the stress of mastication. The possibility of so using the silicate cement for almost all purposes of restoration in cavities of decay is too new and too broad a field for me to consider here.

### CAVITY PREPARATION.

Cavities should be prepared with all the care used for the insertion of gold, but with a slight modification in form. While the cement has some adhesive qualities, it is well always to depend only on a retentive shape, hence the usual undercuts, though rather shallow ones, should be made. The enamel margins should be beveled as little as possible consistent with the proper protection of the enamel rods. The nearer the cavity walls approach the perpendicular to the bottom of the cavity the better, as a feather-edge of cement must always be weak and liable to chip. In a deep-seated cavity where there is danger of injuring the pulp by the pressure necessary to insert this filling, a rigid floor should be made to cover the pulpal wall by flowing over it a good oxyphosphate, the dentin having been first rendered sterile and put in safe condition. While an exposed or nearly exposed pulp may be strangulated under the pressure of packing the filling, I have seen no case, nor heard of an instance, in which there was evidence of chemical irritation caused by the contact of the filling with the dentin.

When the cavity has been perfected, it should be finally cleansed and dried with alcohol. Absolute dryness of the parts near the filling must then be maintained for at least twenty, preferably thirty minutes, and the application of the rubber dam is advised in all cases.

### MANIPULATION.

The proper shade should of course be determined while the tooth is wet, by means of the shade guide, or trial mixes formed into points. The powder or powders to be blended are selected and placed on an agate slab, beside the proper amount of liquid. With an agate or bone spatula carry to the liquid a mass of the powder of about equal bulk. Mix with a light rapid motion, then add small quantities of the r-e maining powder—being sure that a given quantity is incorporated before taking the next—and continue this deft but thorough mixing in the most expeditious manner, not spreading it over a wide surface nor wasting time in scraping the slab. As the mix becomes thicker and finally tough and stiff, some pressure and a kind of patting motion is needed to work the mass into a uniform consistency in which no dry particles of powder remain. The average mix should be a little softer than warm gutta-percha, and it should be carried in pellets and packed rapidly and accurately into all parts of the cavity, great care being taken to get perfect adaptation at the margins and to condense the entire surface of the filling while it is still soft. This can best be done by means of ivory or tortoise-shell burnishers, supplemented by celluloid strips and disks, all lightly greased with vaseline. Surplus material may be trimmed away with keen blades. The ideal result is the perfectly condensed and burnished filling, so nicely finished at the margins as to require no grinding or further shaping. But as it demands almost infinite skill and dispatch not to be caught by the setting, most fillings when set have to be trimmed and finished with disks and strips. In this way absolutely perfect margins may be obtained, and the high polish nearly restored by fine disks. After twenty-five minutes the filling should be quite hard, when all vaseline may be removed by alcohol, and hot paraffine or sandarac varnish spread over the filling and adjoining parts. If the filling is an approximal one, the dam may then be so stretched as to expose the rubber septum between the teeth; this is cut, and the dam removed.

A minimum amount of vaselin should be used on the instruments in finshing, and great care should be taken to prevent its being mixed into the filling, or getting between the filling and the cavity margins. Of course all instruments used for packing must be absolutely clean and free from the slightest trace of grease.

Tortoise-shell points are now made for packing the cement, and are suited for large accessible cavities. I confess that I have encountered no bad results in using polished steel pluggers, though their use is tabooed by the manufacturers.

### CONCLUSIONS.

It is still a common experience in daily practice, while examining the upper incisors of an elderly patient to happen unawares upon a cleverly concealed gold filling, smooth and dense and bright like an old coin, surrounded by perfect and highly polished cavity margins. Upon inquiry you are told that that was the work of some operator now famous in our history, and that there it has stood through the vicissitudes of fifty years. It would be idle to compare the broad usefulness of modern methods in dental practice with the primitive efforts of that early but determined workman, yet in this given instance it is impossible to escape the question, How will our finest fillings in fused or plastic porcelain compare with such work after they have stood the test of time? It is probable that no one as yet dares to be very sanguine in such a speculation. The inlay, however, or the silicate filling which serves its purpose for ten years and is then easily renewed, while requiring constant watching, may give the patient in the course of a lifetime greater satisfaction than a poorly concealed or conspicuous gold filling. Most persons of refinement will prefer to guard a transient perfection rather than undergo, for the sake of permanence, a lasting disfigurement. These admirable specimens of ancient gold fillings are the occasional exceptions among the unsightly bulwarks of gold we now wish to eliminate, built by the same operators.

In comparison with porcelain, the writer doubts if, in cases of large restorations, silicates will ever give the same results in the blending of shades, and translucency. It is to be particularly commended in small approximal cavities. —*Cosmos.* See Ames' discussion page 463 April *Cosmos.*

